# Surgical approaches for the treatment of posterior malleolar fracture: which one to choose?

**DOI:** 10.1530/EOR-2025-0157

**Published:** 2026-06-01

**Authors:** Enrique Fernández-Rojas, Lily Fletcher, Mario Herrera-Pérez, Jesús Vilá-Rico

**Affiliations:** ^1^ Foot and Ankle Group, Traumatology and Orthopedics Unit, Las Higueras Hospital, Talcahuano, Chile; ^2^ Universidad Católica de la Santísima Concepción, Concepción, Chile; ^3^ Traumatology and Orthopedics Unit, Ribera Povisa Hospital, Vigo, Spain; ^4^ Unidad de Pie y Tobillo, Servicio de Traumatología y Cirugía Ortopédica, Hospital Universitario de Canarias, Santa Cruz de Tenerife, España; ^5^ Escuela de Medicina, Universidad de La Laguna, San Cristóbal de La Laguna, España; ^6^ Traumatology and Orthopedics Unit, University Hospital October 12, Madrid, Spain; ^7^ Complutense University of Madrid, Madrid, Spain; ^8^ Department of Orthopaedic Surgery and Traumatology, Quirónsalud Hospital, Madrid, Spain

**Keywords:** ankle fracture, trimalleolar fracture, posterior malleolus, treatment, posterior approach

## Abstract

- Evidence suggests that posterior approaches may be superior to the anterior percutaneous approach in terms of reduction quality and functional outcomes.
- There is no consensus regarding the definition of the posteromedial approach and its modifications, which can cause confusion when evaluating outcomes.
- Several variables must be considered when choosing a surgical approach, with particular importance given to the morphology of the posterior malleolus fracture, the presence of a complex fibula fracture, and the presence of Tillaux–Chaput or Le Fort–Wagstaffe fractures.
- This article provides an overview of approaches to posterior malleolus fractures and presents recommendations for approach selection in specific clinical scenarios.

## Introduction

The treatment goal for a posterior malleolus fracture (PMF) is to achieve an anatomical reduction in order to restore the articular surface of the distal tibia and syndesmotic stability, if compromised. Numerous surgical approaches have been described for this purpose, including anterior percutaneous, posterior percutaneous, posterolateral, and posteromedial approaches and their modifications, and the Achilles tendon-splitting approach (1).

When comparing the anterior percutaneous approach with posterior approaches, evidence shows that the former results in poorer reduction quality (2, 3), greater need for syndesmotic fixation (4), and poorer functional outcomes (5, 6). Despite this, the anterior percutaneous approach has been recommended for Bartoníček type IV PMFs with an articular step-off less than 2 mm, no impaction of the tibial articular surface, and no intercalary fragments (7, 8). For this approach, it is suggested to control the reduction through the fibular fracture, whenever possible (8, 9, 10). The percutaneous approach has also been recommended for minimally displaced PMFs in patients with soft tissue damage, poor bone quality, or poorly controlled diabetes mellitus (11, 12). Another valid option in these cases is the posterior percutaneous approach, which has been reported by several authors (12, 13, 14). The risk of injury to neurovascular and tendon structures must be taken into consideration when performing purely percutaneous approaches.

### Posterolateral approach

The posterolateral approach can be performed in the prone or lateral decubitus position (15). Elevation of the operated ankle to prevent the contralateral limb from interfering with intraoperative lateral fluoroscopy imaging is advised. The dissection between the *flexor hallucis longus* (FHL) and the peroneal tendons is used to access the PMF and the lateral malleolus fracture. However, a second window has been described to access the fibula laterally by retracting the peroneal tendons medially. This allows for lateral plate placement on the fibula, which is more practical for most orthopedic surgeons (8, 16). To decide on the position of the fibular plate, it should also be considered that the posterior plate provides greater stability in biomechanical studies (17, 18).

In a retrospective study of 164 patients, Herbosa *et al.* reported that using a single window yields a better range of motion at 12 months post-operatively and fewer wound complications compared to the two-window approach (19). Anatomical structures at risk in this approach include the sural nerve and the peroneal artery. If the incision begins at the fibular tip, the sural nerve crosses the approach at an average of 3.4 cm (range: 2.7–4.5 cm) from the distal end (20). Typically, a communicating branch between the peroneal artery and the posterior tibial artery must be coagulated to access and reduce the posterior malleolus. However, due to the many anatomical variations of the peroneal artery, careful dissection is essential (21).

### Posteromedial approach

There is significant variability in the descriptions of the posteromedial approach and its modifications. Various intermuscular corridors can be used to access the PMF, listed from medial to lateral: the corridor between the medial malleolus and the posterior tibial tendon (PTT) (22, 23, 24, 25), the corridor between the PTT and the flexor digitorum longus (FDL) (16, 24, 26, 27, 28, 29), and the corridor between the FDL and the FHL, with two possible windows, displacing the neurovascular bundle medially (1, 16, 26, 30, 31, 32) or laterally (1, 30, 33, 34). Finally, the corridor between the FHL and the Achilles tendon can also be used (22, 23, 24, 35). All these approaches have been described in both prone and supine positions, highlighting a clear lack of consensus.

### Proposal for new nomenclature for posteromedial approaches

Given the variability in definitions of the posteromedial approach and its modifications, we propose a nomenclature to unify concepts and facilitate comparison of results from different studies.

The approach using the corridor between the medial malleolus and the PTT in the supine position will be termed ‘medial posteromedial approach’ (M-PM). The approach between the PTT and the FDL tendon will be termed ‘posteromedial approach’ (PM), as it is the first known posteromedial approach described in the literature (28). The approach using the corridor between the FDL and FHL tendons will be called the ‘modified posteromedial approach’ (mPM), which can be performed using two distinct windows by displacing the neurovascular bundle either laterally (1, 16, 26, 30, 31, 32) or medially (1, 16, 26, 30, 31). Finally, the approach using the corridor between the FHL and the Achilles tendon will be referred to as the ‘lateral posteromedial approach’ (L-PM). This last concept is new and is proposed because it uses the most lateral intermuscular corridor accessible from the posteromedial route, allowing differentiation from the previously described approaches (Table 1).

**Table 1:** Proposed nomenclature for posteromedial ankle approaches.

Approach	Description
Posteromedial approach	Between PTT and FDL
Modified posteromedial approach	Two windows:
	- Between FDL and FHL, retracting the neurovascular bundle laterally - Between FDL and FHL, retracting the neurovascular bundle medially
Medial posteromedial approach	Between the medial malleolus and the PTT
Lateral posteromedial approach	Between FHL and the Achilles tendon

PTT, posterior tibial tendon; FDL, flexor digitorum longus; FHL, flexor hallucis longus.

### Guide for selecting posterior approaches

To provide guidance for selecting surgical approaches to treat PMF, we define three key injury-related variables. The first is PMF morphology, as defined by the Bartoníček and Rammelt or Mason classification (36, 37). Another variable to consider is the presence of a complex fibula fracture, defined as comminuted fractures or fractures in the middle third of the fibula. The third variable is the presence of anterolateral tubercle fractures of the distal tibia (Tillaux–Chaput fragment) or anteromedial tubercle fractures of the fibula (Le Fort–Wagstaffe fragment), as these indicate anterior syndesmotic injury (38, 39, 40) (Table 2).

**Table 2:** Proposal for choosing surgical approaches in PMFs.

	+/− Non-complex fibula FX	Complex fibula FX (A)	Chaput/Le Fort FX (B)	(A) + (B)
PMF B-R types II and IV or Mason types 2A and 3	PL	L-PM + lateral	PL + AL (PC)	L-PM + lateral (PC)
	L-PM + lateral	PL (2 windows)	L-PM + AL (PC)	PL (2 windows) + AL (PC)
PMF B-R type III or Mason type 2B	PM + lateral	PM + lateral	M-PM + AL	M-PM + lateral
	mPM + lateral	mPM + lateral	PM + AL (PC)	PM + lateral (PC)
	M-PM + lateral	M-PM + lateral	mPM + AL (PC)	mPM + lateral (PC)
	L-PM + lateral	L-PM + lateral	L-PM + AL (PC)	L-PM + lateral (PC)
	PL + PM (double approach)			

FX, fracture; PMF, posterior malleolus fracture; B-R, Bartoníček and Rammelt; PL, posterolateral approach; PM, posteromedial approach; L-PM, lateral posteromedial approach; M-PM, medial posteromedial approach; mPM, modified posteromedial approach; PC, position change.

The combination of the latter two variables leads to four major clinical scenarios, which are further subdivided based on PMF morphology.

Even though only these variables were considered to simplify the algorithm, it is clear that other factors must also be taken into account when choosing an approach. One such situation is acute medial instability. When ligament repair is indicated, the procedure may be more easily performed with the patient in the supine position, similarly to cases involving Le Fort–Wagstaffe or Tillaux–Chaput fractures (38, 39). However, it may also be performed with the patient in the prone position, with the knee flexed to 70–90°, or even in the floppy lateral position, which may be particularly useful when a posterolateral approach is also required (41).

Another is syndesmotic instability – while syndesmotic fixation can be done in the prone position, assessing the reduction quality *in situ* is easier with the patient in the supine position. It is also important to consider the presence of a medial malleolus fracture, which, like the aforementioned cases, is more easily reduced in the supine position. If the patient is in the prone position, we recommend fixing the medial malleolus by flexing the knee to 70–90°, which allows assessment of the reduction using the anterior Harty notch as a reference – our usual marker for confirming an anatomic reduction.

### Scenario 1: isolated posterior malleolus fracture with indication for fixation or associated with a non-complex fibula fracture

For isolated Bartoníček type II and IV or Mason 2A and 3 fractures with indication for fixation, or those associated with a non-complex fibula fracture, a posterolateral approach or a lateral posteromedial approach can be used, as the latter allows visualization of 71% of the posterior surface of the tibia, particularly the posterolateral region (42).

**Figure 1:**
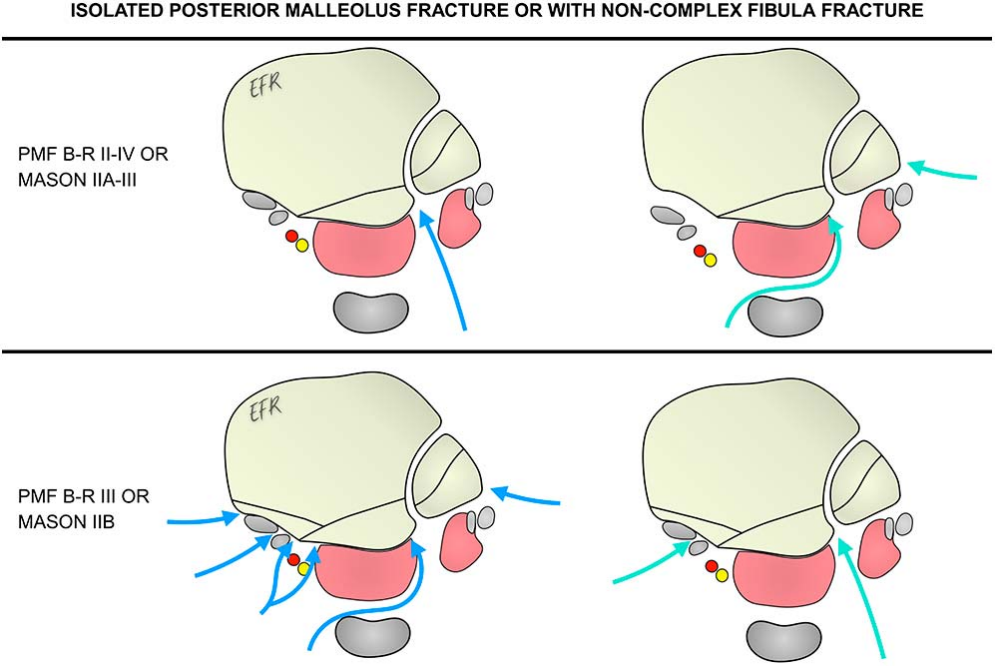
Approach options for a PMF with surgical indication, either isolated or associated with a non-complex fibula fracture. PMF, posterior malleolus fracture; B-R, Bartoníček and Rammelt.

In contrast, for Bartoníček type III or Mason 2B fractures, posteromedial approaches or their modifications are suitable, as they provide better access to the posteromedial fragment (43, 44, 45). Additionally, when posteromedial fragments are present, it is recommended to begin reduction with that fragment and then proceed to reduce the posterolateral fragment (22) (Fig. 1).

If a non-complex fibula fracture is present, it can be reduced and fixed through the same posterolateral approach. However, if a posteromedial approach or its modifications are used, a lateral ankle approach is recommended to fix the fibula.

### Scenario 2: posterior malleolus fracture associated with a complex fibula fracture

**Figure 2:**
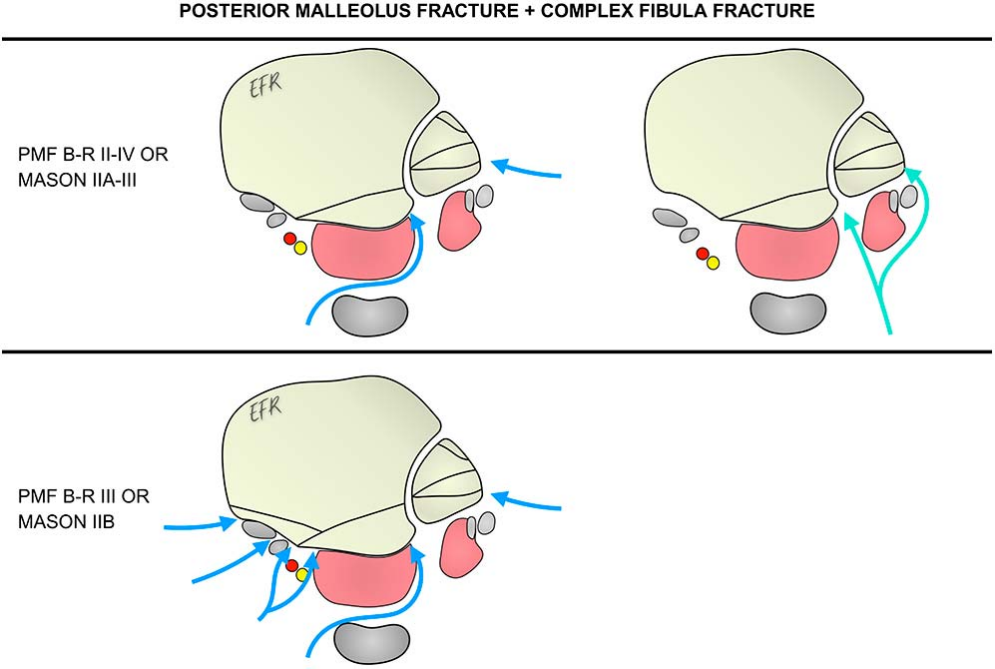
Approach options for a PMF associated with a complex fibula fracture. PMF, posterior malleolus fracture; B-R, Bartoníček and Rammelt.

The presence of a complex fibula fracture makes it advisable to approach the fibula via a lateral approach or, alternatively, a two-window posterolateral approach, retracting the peroneal tendons medially and accessing the fibula laterally (Fig. 2).

### Scenario 3: posterior malleolus fracture associated with a Tillaux–Chaput or Le Fort–Wagstaffe fracture

Reduction of Chaput or Le Fort fractures may be easier in the supine position, although it can also be performed in the prone position, as mentioned previously. Therefore, three options arise: performing the entire procedure with the patient in either the prone or supine position, or changing the patient’s position during surgery in order to perform an anterolateral approach.

**Figure 3:**
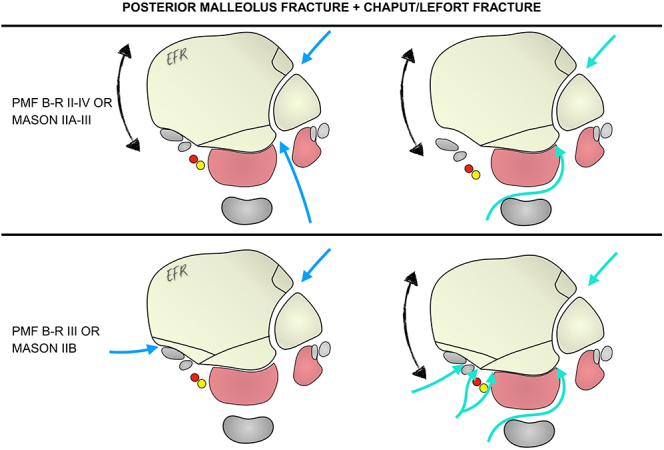
Options for surgical approaches for a PMF associated with a Tillaux–Chaput or Le Fort–Wagstaffe fracture. PMF, posterior malleolus fracture; B-R, Bartoníček and Rammelt.

An alternative to the anterolateral approach is a lateral approach that curves anteriorly at its distal end, allowing access to the aforementioned fragments. However, this must also be performed with the patient in the supine position (Fig. 3).

### Scenario 4: posterior malleolus fracture associated with both a complex fibula fracture and a Tillaux–Chaput or Le Fort–Wagstaffe fracture

**Figure 4:**
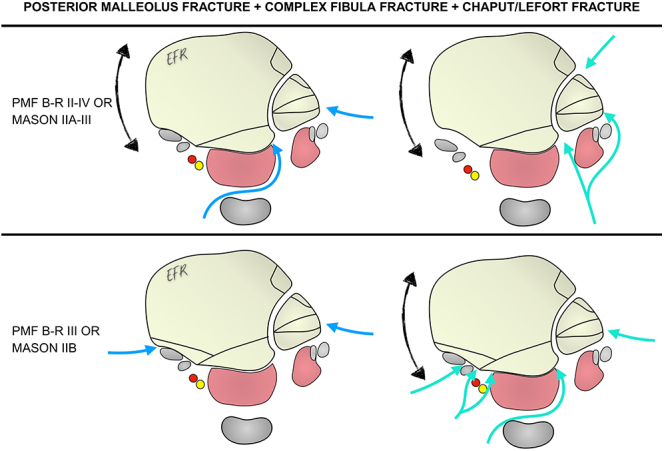
Options for surgical approaches for a PMF associated with a complex fibula fracture and a Tillaux–Chaput or Le Fort–Wagstaffe fracture. PMF, posterior malleolus fracture; B-R, Bartoníček and Rammelt.

The presence of both variables complicates surgical approach selection, as it requires lateral exposure of the fibula while also making supine positioning desirable at some stage of the procedure to facilitate fixation of the Chaput or Le Fort fragments. It should be noted that other authors have also described the possibility of fixing all these fractures with the patient in the prone position (38) (Fig. 4).

## Discussion

Some authors have previously made recommendations regarding the selection of surgical approaches for PMFs. Rammelt and Bartoníček, based on their classification of these injuries, recommended the posterolateral approach for type II and IV PMFs; the posteromedial, posterolateral, or dual approach for type III PMFs; and the anterior percutaneous approach for type IV PMFs without articular step-off or intercalary fragments (7, 33, 37, 46).

On the other hand, Mason *et al.*, based on their own classification, recommended the posterolateral approach for type 2A PMFs, the posteromedial or dual approach for type 2B PMFs, and the posteromedial approach for type 3 (22). While these guidelines are useful, they only consider the morphology of the posterior malleolus, overlooking many other associated injuries that should influence the choice of approach. Arrondo and Joannas published a practical guide for the surgical management of these fractures, adding important variables to consider, such as the presence of lateral malleolus fractures, medial malleolus fractures, and/or Tillaux–Chaput or Le Fort–Wagstaffe fragments. Based on the combination of these injuries, they created different scenarios and recommended specific approaches for each one (26). As authors, we believe it is important to have more than one approach option for each clinical scenario, since areas of exposure or soft tissue conditions often dictate the choice of approach. With this in mind, we previously published some therapeutic guidelines based on three key variables (though not the only ones) that we consider fundamental when selecting a treatment strategy (47). These are the following: posterior malleolus morphology, the presence of Chaput or Le Fort fragments, and the presence of complex fibula fractures, which we define as comminuted or involving the middle third of the fibula. This last variable had not been considered in the recommendations provided by other authors (16, 26, 35). Based on these factors, we proposed a primary approach option and alternative options for each clinical scenario (47).

The current guide introduces some modifications, such as removing the recommendation of a single first-choice approach and instead presenting all possible approach options, as we believe multiple approaches may be equally suitable for a given clinical scenario. The choice should depend on soft tissue condition and surgeon’s experience.

Additionally, considering that the classifications by Bartoníček *et al.* and Mason *et al.* are widely used, we included both in the description of PMF morphology. By incorporating the types described by Mason *et al.*, we broaden the guide’s utility for surgeons more familiar with that classification. The newly proposed nomenclature for the variants of the posteromedial approach, along with the introduction of the ‘lateral posteromedial approach’ concept, may help in standardizing terminology and avoiding the confusion caused by the numerous modifications of the posteromedial approach.

## Conclusion

PMFs are complex injuries, as they are usually accompanied by other associated lesions. When selecting the surgical approach, it is important to consider not only the fracture’s morphology but also any associated bony or ligamentous injuries, patient characteristics and comorbidities, and the surgeon’s experience.

Even though certain guidelines are provided to assist in the decision-making process for choosing an approach, we must keep in mind that there is more than one correct way to surgically approach the same PMF.

## ICMJE Statement of Interest

The authors declare that there is no conflict of interest that could be perceived as prejudicing the impartiality of the work reported.

## Funding Statement

This research project did not receive any specific grants from public sector agencies, commercial sector, or non-profit entities.
